# Effects of Avatar Perspective on Joint Excursions Used to Play Virtual Dodgeball: Within-Subject Comparative Study

**DOI:** 10.2196/18888

**Published:** 2020-08-19

**Authors:** Susanne M van der Veen, Alexander Stamenkovic, Megan E Applegate, Samuel T Leitkam, Christopher R France, James S Thomas

**Affiliations:** 1 Virginia Commonwealth University Richmond, VA United States; 2 Harvard University Boston, MA United States; 3 McGill University Montreal, QC Canada; 4 Ohio University Athens, OH United States

**Keywords:** virtual reality, avatar perspective, reaching, joint excursion, exergaming, exercise rehabilitation, head mounted display

## Abstract

**Background:**

Visual representation of oneself is likely to affect movement patterns. Prior work in virtual dodgeball showed greater excursion of the ankles, knees, hips, spine, and shoulder occurs when presented in the first-person perspective compared to the third-person perspective. However, the mode of presentation differed between the two conditions such that a head-mounted display was used to present the avatar in the first-person perspective, but a 3D television (3DTV) display was used to present the avatar in the third-person. Thus, it is unknown whether changes in joint excursions are driven by the visual display (head-mounted display versus 3DTV) or avatar perspective during virtual gameplay.

**Objective:**

This study aimed to determine the influence of avatar perspective on joint excursion in healthy individuals playing virtual dodgeball using a head-mounted display.

**Methods:**

Participants (n=29, 15 male, 14 female) performed full-body movements to intercept launched virtual targets presented in a game of virtual dodgeball using a head-mounted display. Two avatar perspectives were compared during each session of gameplay. A first-person perspective was created by placing the center of the displayed content at the bridge of the participant’s nose, while a third-person perspective was created by placing the camera view at the participant’s eye level but set 1 m behind the participant avatar. During gameplay, virtual dodgeballs were launched at a consistent velocity of 30 m/s to one of nine locations determined by a combination of three different intended impact heights and three different directions (left, center, or right) based on subject anthropometrics. Joint kinematics and angular excursions of the ankles, knees, hips, lumbar spine, elbows, and shoulders were assessed.

**Results:**

The change in joint excursions from initial posture to the interception of the virtual dodgeball were averaged across trials. Separate repeated-measures ANOVAs revealed greater excursions of the ankle (*P*=.010), knee (*P*=.001), hip (*P*=.0014), spine (*P*=.001), and shoulder (*P*=.001) joints while playing virtual dodgeball in the first versus third-person perspective. Aligning with the expectations, there was a significant effect of impact height on joint excursions.

**Conclusions:**

As clinicians develop treatment strategies in virtual reality to shape motion in orthopedic populations, it is important to be aware that changes in avatar perspective can significantly influence motor behavior. These data are important for the development of virtual reality assessment and treatment tools that are becoming increasingly practical for home and clinic-based rehabilitation.

## Introduction

Virtual reality (VR)-based interventions hold great potential for rehabilitation, as they can be used to both assess motor coordination and elicit specific movements in a manner that is simultaneously engaging and therapeutic. Further, visual stimuli can be easily presented in VR and manipulated in real-time, providing insight into the neural mechanisms underpinning sensorimotor control of movement. While VR systems are becoming more affordable and readily available for in-home rehabilitation applications, the effects of different avatar perspectives on motor behavior are poorly understood and need to be studied to optimize interventions. For example, the use of home devices such as the Kinect sensor, which tracks and presents an avatar in a third-person perspective, may result in very different motor behavior when compared to the same tasks being presented from the first-person perspective.

Virtual reality has been used to shape motion in orthopedic [1-3] and neurologic patient populations [4-6], with reports showing significant effects on pain relief, joint mobility and motor function [7]. However, the vast differences in methodology, especially concerning visual display type, avatar perspective, and level of gameplay immersion, make it difficult to draw broad conclusions about which features are driving the efficacy of VR treatments [7]. Visual environments in VR can present 3-dimensional (3D) images across a variety of display devices, including head mounted-displays (HMD) [6] and 3D televisions (3DTV) [8,9]. The different methods used to present visual scenes can affect how the virtual environment is perceived and thus can influence not only motor behavior [8] but pain responses as well [10].

While Thomas and colleagues have shown that avatar perspective influences joint excursions in full-body reaching tasks [9] as well as during VR gameplay [8], it is unknown if the differences in joint excursions were driven by avatar perspective (first- or third-person) or by display type (HMD or 3DTV). Ustinova et al [11] reported that individuals reached further and preferred tasks when the camera perspective was oriented at angles from 45 to 77.5 degrees relative to the location of the avatar as compared to the camera oriented at zero degrees (ie, directly behind the avatar) [11]. The increased segment displacement was accompanied by a slightly larger displacement of the whole-body center of mass, yet these reaches were always made to varying degrees in the third-person avatar perspective.

To date, no study has compared the effect of the avatar perspective alone on the apportionment of joint excursions in VR tasks while keeping the display type constant. This study was designed to determine the effects of avatar perspective (ie, first- versus third-person) on joint excursions of healthy participants engaged in full-body movements during a VR dodgeball game presented on an HMD. Based on existing studies [8,9,11], we predicted that joint excursions would be greater in the first person versus third-person perspective.

## Methods

### Recruitment

We recruited 29 healthy young adults (15 male, 14 female) aged 18-35 years (mean ± SD, 23 ± 1.62 years, range 20-28 years). Exclusion criteria included a history of a low back injury, low back pain within the last 6 months, and any orthopedic, neurological, or visual impairment that would prevent participation. This study was approved by the Institutional Review Board of Ohio University, and written informed consent was obtained at the beginning of the session.

### Instrumentation

Movement of light-reflective marker clusters attached to the head, upper arms, forearms, hands, trunk, pelvis, thighs, shanks, and feet was tracked using a 10-camera Vicon Bonita system sampling at 100 Hz. This optoelectric-based kinematic system can track the 3D coordinates of light reflective marker clusters attached to the participant with a spatial resolution of 0.1 mm. The time-series joint angle data were derived from the 3D segment coordinate data using an Euler angle sequence of (1) flexion-extension, (2) lateral bending, and (3) axial rotation using MotionMonitor software (The MotionMonitor) [12]. Joint excursions were defined as the change in joint angle from initial standing posture to posture at interception of launched virtual balls.

### Procedures

**Figure 1:**
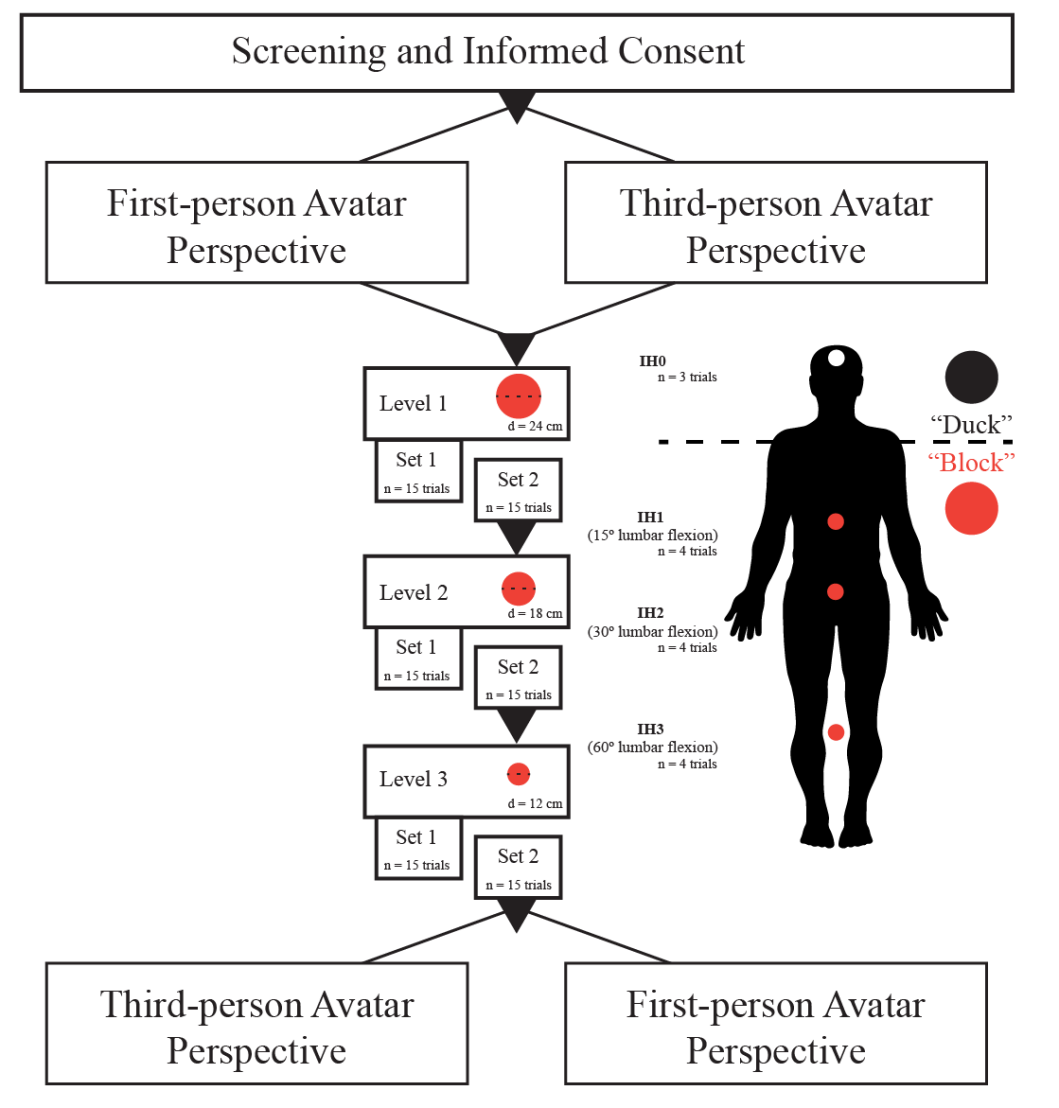
A flow chart of the recruitment, randomization, and dodgeball gameplay. The red dots representing impact heights are solely for visualization.

The study employed a within-subjects design, in which participation consisted of gameplay during two virtual sessions of dodgeball. Each session involved gameplay presented in either the first- or third-person perspective. A first-person perspective was created by placing the center of the displayed content at the bridge of the participant’s nose, while a third-person perspective was created by placing the camera view at the participant’s eye level and set 1 m behind the participant avatar. The order of avatar perspective was randomized and counterbalanced such that half the study cohort began with gameplay using a first-person perspective, and half began with a third-person perspective (see Figure 1). During gameplay, participants competed against four virtual opponents, and the object was to block or avoid virtual balls launched by the four opponents. The intended impact heights of the launched virtual balls were identical between the two avatar perspectives (see Gameplay section for a description of impact height determination). Participants earned game points (which were associated with actual cash rewards) by either successfully blocking the virtual balls (see Figure 1, "Duck") or, in the case of certain colored balls, avoiding contact with them.

### Virtual Environment

Vizard software (WorldViz) was used to develop the virtual environment and control all presented graphics and audio stimuli, including the opposing team’s avatars. The six degrees of freedom kinematic data from the clusters of light reflective markers placed on the participant was streamed to the game environment at 100 Hz using Vicon Tracker software, allowing for near real-time presentation of the participant’s avatar (39 ms latency). The MotionMonitor software was used to control bidirectional communication with Vizard, set game parameters and target locations, and record all kinematic data during the experimental testing session. Participants viewed their avatar from a first- or third-person perspective via an HMD (Oculus Rift Developers Kit 2, Oculus). The HMD display provided a 90° horizontal field of view, and the refresh rate was fixed at 75 Hz/eye.

### Gameplay

**Figure 2:**
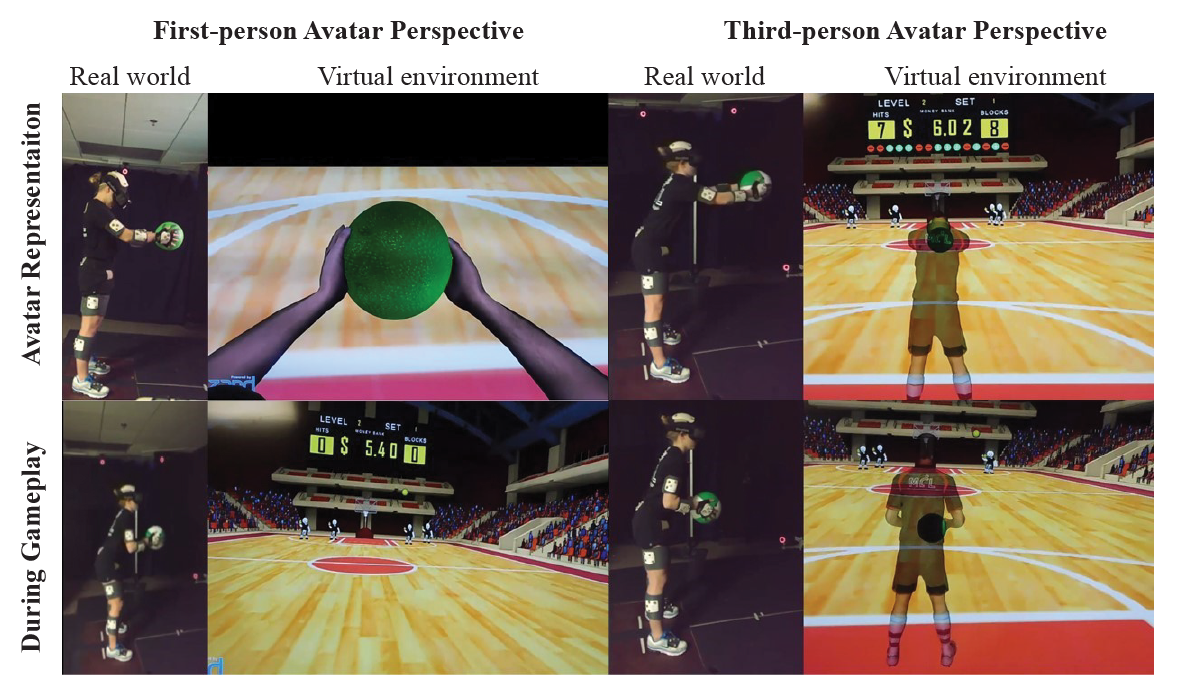
Participant instrumented and engaged in virtual dodgeball using a head-mounted display. Differences in the representation of the avatar in the virtual environment (upper panels) and during gameplay (bottom panels) are shown for both the first-person (left panels) and third-person perspectives (right panels).

**Figure 3:**
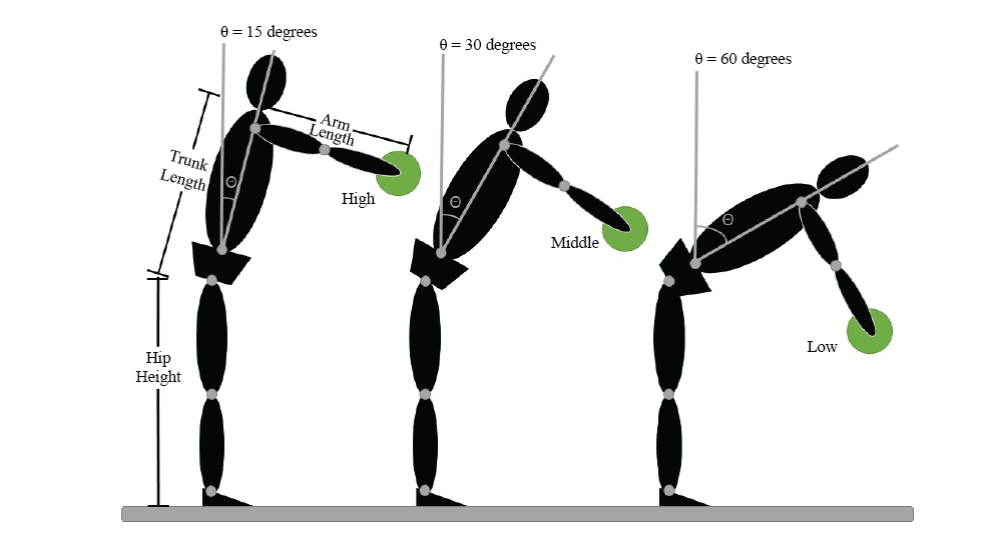
Methods for computing location of the impact heights (IH1: highest; IH3: lowest) of the launched virtual balls for a single game level. Impact heights were tailored for each participant based on their hip height, trunk length, and arm length to varying degrees of lumbar spine flexion.

The game environment was an indoor basketball arena, with the participant positioned at the free-throw line on one side of the court, facing the four virtual opponents positioned on the opposite free-throw line. The opposing players moved 3 m fore-aft and 3m left-right in random order. Virtual balls were launched with a speed of 30 m/s every 3.3 ± 0.3 seconds in a randomized order from each of the four virtual opponents. To indicate the launching of a virtual ball, an opponent would flash either green or yellow 300 ms before release. The color signified whether participants had to block the oncoming ball (green flash, red ball), using the virtual ball co-located with a physical dodgeball in the real world (and held between the participant’s hands), or avoid the ball by “ducking” below it (yellow flash, black ball). This second condition was accompanied by an audible duck quacking sound to emphasize the goal of ducking to avoid the ball. A large scoreboard was positioned at the opposite end of the arena (above the opponents) so that participants could track their performance and cash rewards earned. Additional sound effects included crowd cheering, buzzers, and referee whistles. An instrumented participant engaged in virtual dodgeball with the HMD is shown in Figure 2. A gameplay session consisted of three levels lasting approximately 2 minutes. Each game level consisted of 2 sets with 15 balls in each set. The intended impact locations of 12 of the 15 balls launched were distributed amongst the three impact heights normalized to the participant’s arm length, trunk length, and hip height [8,9,13-16]. For example, during gameplay, the participant could successfully block a virtual ball launched to impact height 1 (ie, IH1, the highest impact height) simply by flexing at the lumbar spine 15° with the elbow fully extended and the shoulder flexed to 90°. In contrast, 60° of lumbar flexion would be required to intercept a virtual ball under the same conditions launched to IH3 (ie, the lowest impact height, see Figure 3). Three balls were launched at each impact height, one to intersect the participant at their midline and one each 20 cm to the left and right of the midline. An additional three balls were launched targeting the head of the avatar to elicit avoidance of the ball by ducking. As balls that required ducking elicited different movement behaviors (ie, object avoidance rather than interception via a reaching motion), these were not investigated in the current study. It is important to note that the order in which each virtual ball was launched at different impact heights was permutated at each round of gameplay. Each level saw a reduction in the size of the dodgeball thrown, starting with a standard-sized dodgeball (diameter: 24 cm) in level 1, reducing across levels (diameter: 18 cm, level 2; diameter: 12 cm, level 3) in order to increase gameplay challenge (see Figure 1). Gameplay performance was updated in real-time and displayed on the virtual scoreboard, with the participant earning progressively higher rewards for each successful block at each level of play (Practice Level = 1¢, Level 1 = 2¢, Level 2 = 5¢, Level 3 = 10¢). Similar to the popular game of dodgeball, success was determined when the oncoming dodgeball was intercepted before contact with the virtual avatar was made.

Conversely, the participant lost cash rewards for each failure to block an oncoming ball from hitting their virtual avatar. Each player started the game with a cash balance on the scoreboard such that if they failed on every launched or presented ball, their cash balance would be zero. The average gameplay session lasted approximately 15 minutes. Following each session, participants rated their overall effort using the NASA Task Load Index (TLX). The NASA TLX is a multidimensional assessment that rates perceived workload to assess system performance [17]. Specifically, participants provide experience ratings of 1 (very low) to 7 (very high) along six dimensions: mental demand, physical demand, temporal demand, performance, effort, and frustration. In the context of assessing the avatar perspective on game success, this measure provides insight into differences in the perceived workload performing nearly identical tasks.

### Data Reduction and Analysis

As gameplay occurred with the dodgeball centered between the left and right hands of the participants, and initial examination of joint excursions was nearly identical for the left and right. Therefore, analyses have been limited to comparisons for the right side only. First, the time-series position vector of the right index fingertip was smoothed using a 41-point fourth-order Savitzky-Golay filter [18]. That is, at each time sample, fourth-order polynomials were fit in the least-squares sense to the data at that point and 20 neighboring samples on each side. The polynomial coefficients were then used to determine velocity. Movement onset was determined from a backward search from peak velocity and defined as the point where velocity was ≤5% peak velocity. Target contact was defined as the point where velocity was ≤5% peak velocity using a forward search from peak velocity. Movement time was determined from movement onset to target contact, as specified above. The change in joint angles (ie, ankle, knee, hip, spine, shoulder, and elbow) and displacement of the whole-body center of mass, based on the combination of segment center of mass [19], along the antero-posterior, mediolateral, and vertical axes were calculated from movement onset to target contact. To determine hand position at target contact, we first calculated the centroid of the hands from the x, y, and z position traces from marker clusters on the left and right hands and adjusted this to the centroid of the left and right ankle joint. We then determined the hand position at target contact for the antero-posterior, mediolateral, and vertical axes.

### Statistical Analysis

An initial power calculation using G*Power 3.19 [28] based on joint excursions observed during pilot testing found that 14 participants were necessary to determine the within-subject effects of the avatar perspective with 80% power, assuming alpha = .05, a correlation between measures of 0 .5, and an effect size of *f* = 0.4 (large effect). As we were primarily interested in the influence of the avatar perspective on movement behavior, we only analyzed trials using the standard dodgeball size. Separate 3-way repeated measures analyses of variance were performed for each dependent measure, with sex as the between-subjects variable, and perspective (first person, third person) and impact height (IH1-IH3) as within-subject variables. Dependent measures included: (1) movement time, (2) hand position at target contact in the antero-posterior, mediolateral, and vertical planes, (3) joint angular excursions of the right ankle, knee, hip, spine, shoulder, and elbow, (4) displacement of the center of mass (ie, antero-posterior, mediolateral, vertical), and (5) success rate for the standard dodgeball size. Post-hoc analyses were performed using the method of least significant differences. Interactions were examined using a simple effects model. The NASA TLX data were analyzed using paired t-tests with Bonferroni correction for multiple comparisons. All statistical analyses were completed in SPSS 22 (IBM).

## Results

### Movement Time

There was no effect of avatar perspective on movement time, but movement time did differ as a function of impact height (*F*_2,27_=9.181, *P*<.001). Specifically, movement time was less for interception of virtual dodgeballs launched to the highest (574 ms ± 40 ms) versus the middle (648 ms ± 27 ms, *P*=.006) and low impact heights (671 ms ± 23 ms, *P*=.002). There were no interactions of perspective by impact height on movement time.

### Hand Position at Ball Contact

Antero-posterior hand position at ball contact was further forward when the participant’s avatar was presented in a first-person (76.7 cm ± 2.2 cm) versus a third-person (69.1 cm ± 2.7 cm) perspective (*F*_1,27_=20.410, *P*<.001). Antero-posterior hand position at ball contact was also influenced by impact height (*F*_2,26_=12.980, *P*<.001). Specifically, participants did not reach as far forward for virtual balls launched to the lowest impact height (68.1 cm ± 2.6 cm) as compared to the middle impact height (75.7 cm ± 2.4 cm, *P*=.010) and the highest impact height (75.0 cm ± 2.2 cm, *P*=.013). There were no interaction effects of perspective and impact height.

**Figure 4:**
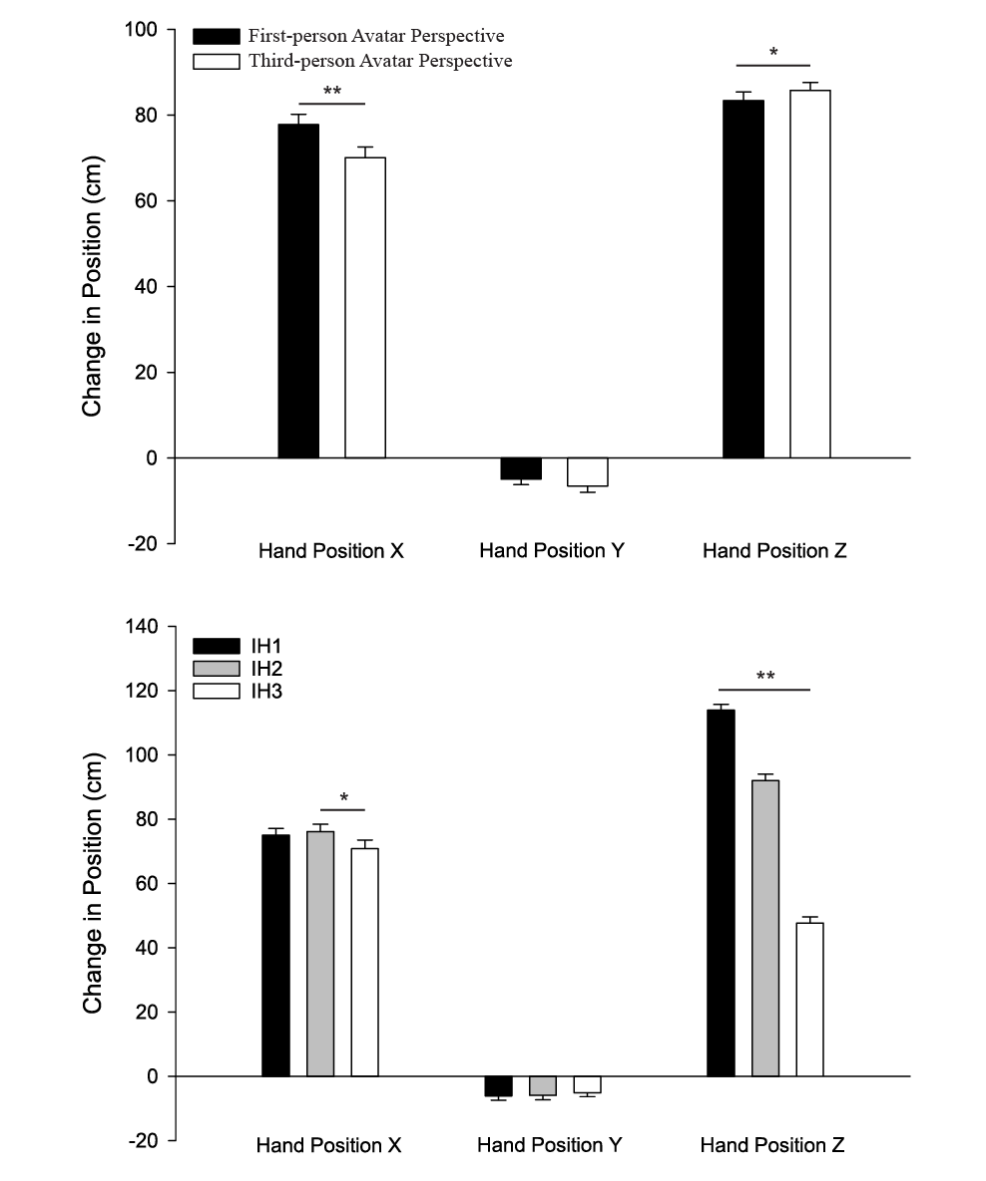
The top panel shows the effect of avatar perspective (first- and third-person) on mean hand position averaged across all three impact heights. The bottom panel shows the effect of target impact height (IH1: high, IH2: middle, IH3: low). Errors bars indicate the standard error of the mean, **P<.001; *P<.05).

There was an interaction of avatar perspective and impact height on vertical hand position at ball contact (*F*_2,26_= 4.237, *P*=.020, see Figure 4). Follow-up analyses revealed that the avatar perspective resulted in differences in vertical hand position at ball contact for the lowest impact height (IH3) (first-person 45.0 cm ± 2.4 cm and third-person 50.4 cm ± 2.1 cm, *P*=.023). Post-hoc analyses revealed that participants had to reach lower to intercept virtual balls launched to the lowest impact height (IH3) (47.7 cm ± 1.9 cm) versus the middle (IH2) (92.0 cm ± 2.0 cm) and highest (IH1) (113.9 cm ± 1.8 cm) impact heights (*P*<.001 for all comparisons). Additionally, an interaction effect of sex and impact height was found (*F*_1,27_=14.158, *P*<.001); post-hoc analysis showed males had lower hand position at ball contact than females for IH1 and IH2 (females, IH1: 117.3 cm ± 2.6 cm, IH2: 93.5 cm ± 2.8 cm; males, IH1: 115 cm ± 2.5 cm, IH2 90.6 cm ± 2.8 cm, *P*=.036 and *P*=.040 respectively).

No significant differences along the mediolateral axis were found.

### Joint Excursions

**Figure 5:**
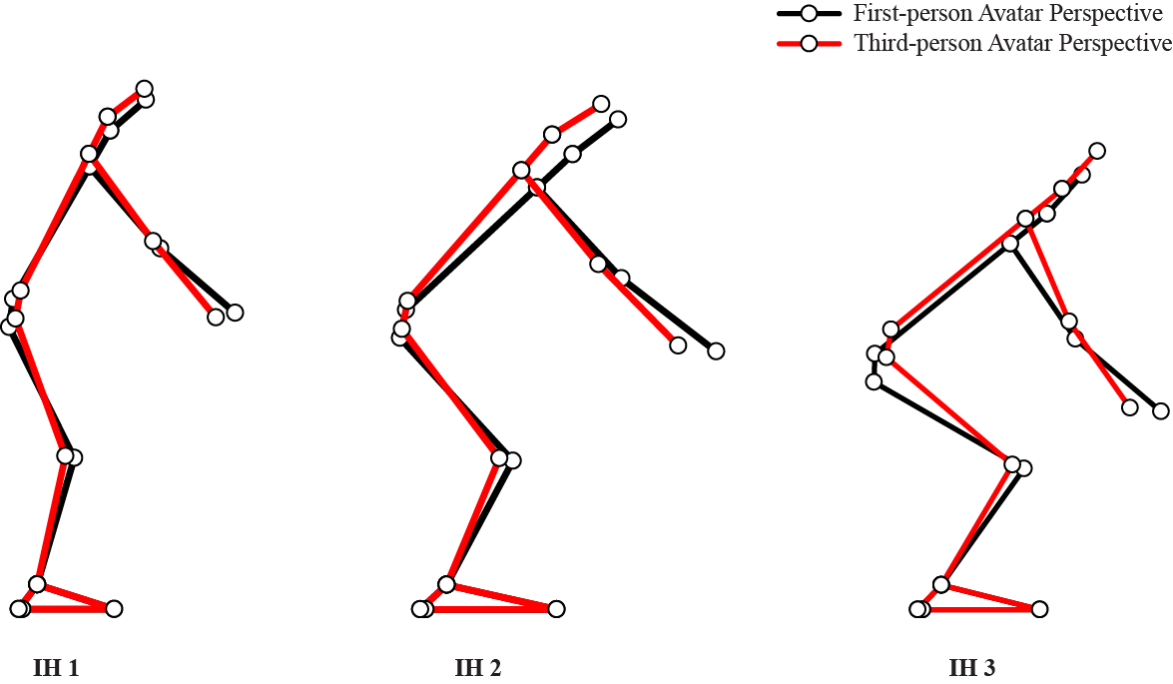
Average effects of first-person avatar perspective versus third-person avatar perspective for IH1 (left panel), IH2 (middle panel), and IH3 (right panel) on the posture adopted at target intercept while playing virtual dodgeball using a head-mounted display.

**Figure 6:**
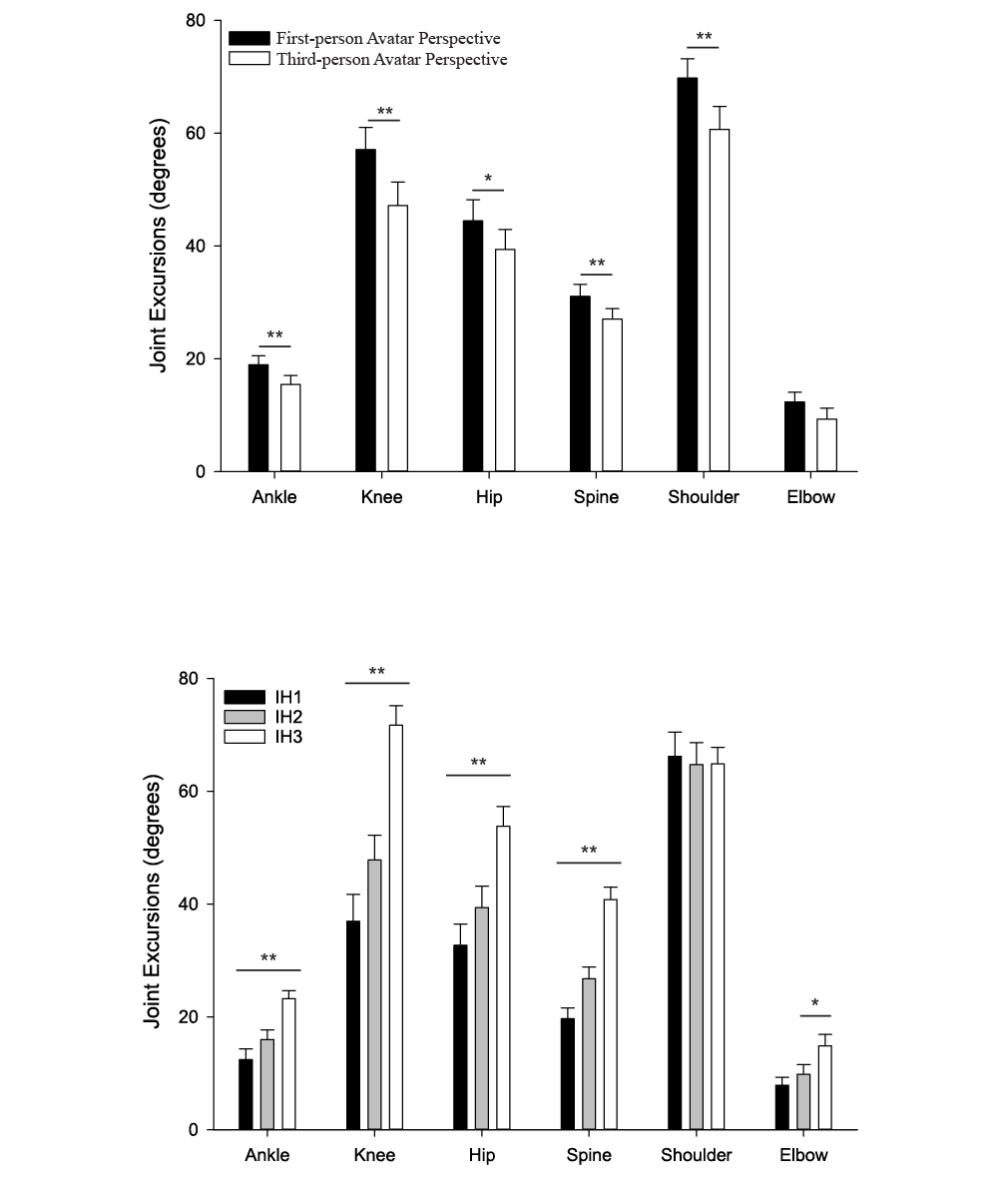
The top panel shows the effect of avatar perspective on mean joint excursions averaged across all three impact heights of the ankle, knee, hip, spine, shoulder, and elbow. The bottom panel shows the effect of target impact height (IH1: high, IH2: middle, IH3: low). Error bars indicate the standard error of the mean, **P<.001; *P<.05.

During gameplay presented in the first-person versus third-person perspective, participants increased excursions of the ankle, F_1,27_=7.570, *P*=.010, knee (F_1,27_=12.797, *P*=.001), hip (F_1,27_=6.899, *P*=.014), spine (F_1,27_=14.515, *P*=.001), and shoulder (F_1,27_=13.223, *P*=.001) (see Figures 5 and 6). However, there was no effect of avatar perspective on elbow excursions across all impact heights.

As expected, most joint excursions increased as a function of impact height. Specifically, participants used greater excursions of the ankle (*F*_2,26_=25.050, p<0.001), knee (*F*_2,26_=51.198, *P*<.001), hip (*F*_2,26_=37.538, *P*<.001), spine (*F*_2,26_=74.462, *P*<.001), and elbow (*F*_2,26_=3.685, *P*=.039), from the highest to the lowest impact height. However, there was no effect of impact height on shoulder excursions.

An interaction between sex and perspective was found for elbow flexion (*F*_1,27_=5.468, *P*=.027), where males showed greater elbow flexion in first-person avatar perspective (11.7° ± 3.0°) than in third-person avatar perspective (10.8° ± 4.7°, *P*=.005).

### Center of Mass Displacement

**Figure 7:**
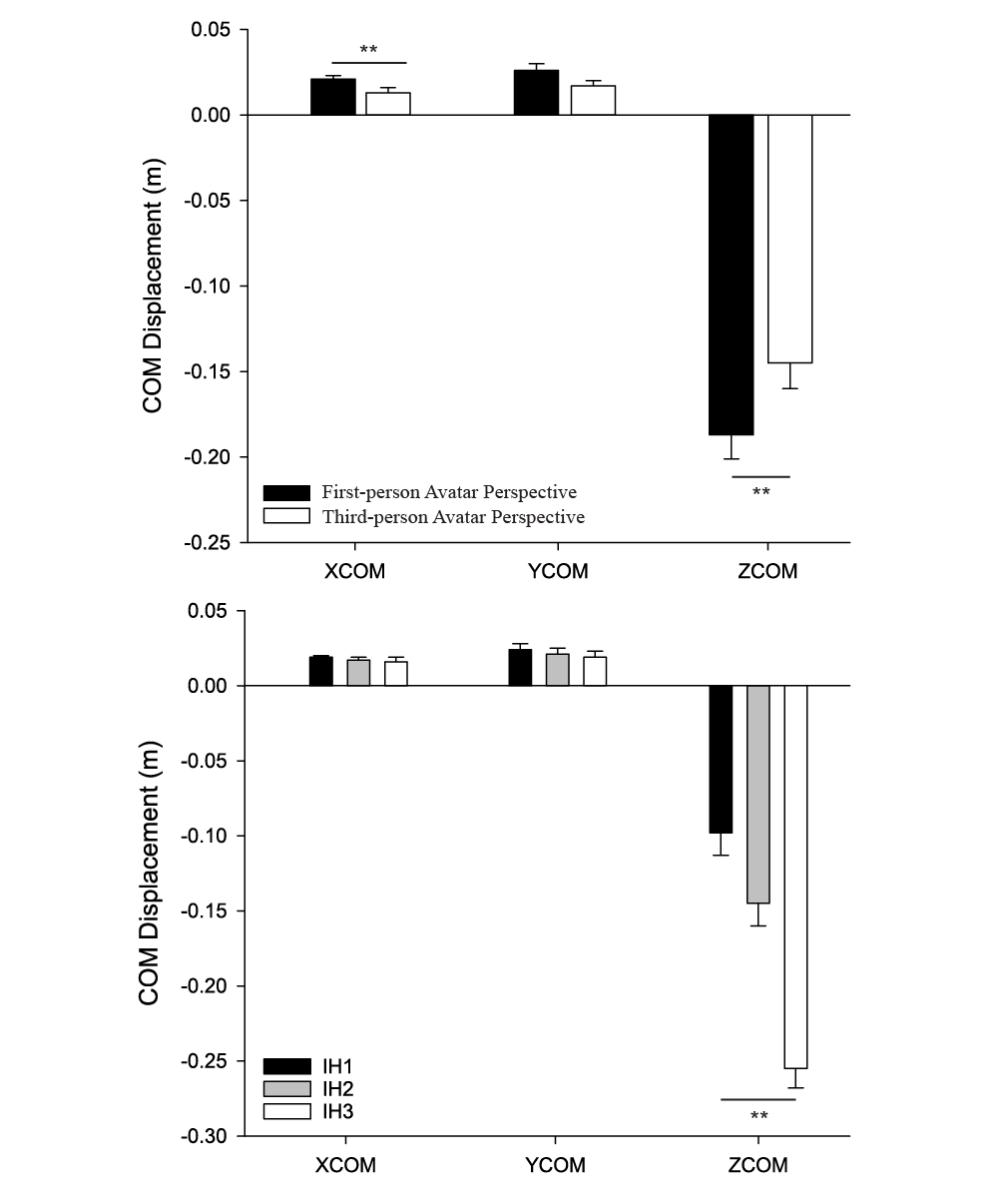
Effects of avatar perspective (top panel) and : predicted interception heights (bottom panel) on mean COM excursions in the antero-posterior (XCOM), mediolateral (YCOM), and vertical (ZCOM) planes. Error bars represent the standard error of the mean **P<.001.

Participants had greater forward displacement (along the antero-posterior axis) of their center of mass while playing in a first-person (2.1 cm ± 0.3 cm) versus third-person (1.2 cm ± 0.3 cm) perspective (*F*_1,27_=16.941, *P*<.001). Impact height also affected antero-posterior displacement of the center of mass (*F*_2,26_=5.987, *P*=.007), with post-hoc analyses revealing that antero-posterior center of mass displacement was greatest for high (IH1 1.9 cm ± 0.2 cm) versus the middle (IH2 1.7 cm ± 0.3 cm, *P*=.034) and low (IH3 1.5 cm ± 0.3 cm, *P*=.004) impact heights (see Figure 7).

There was an interaction of avatar perspective by impact height on vertical displacement (*F*_2,26_=17.405, *P*<.001). Follow-up analyses revealed that vertical center of mass displacement was greater when the avatar was presented in first versus the third person for all target heights (IH1 *P*<.001; IH2 *P*<.001; IH3 *P*=.002), but the magnitude of the difference was least for the lowest impact height.

Mediolateral displacement was greater in the first-person perspective (2.8 cm ± 0.5 cm) versus the third-person perspective (1.6 cm ± 0.4 cm), *F*_1,27_=11.432, *P*=.045. Impact height also affected vertical displacement, *F*_2,26_=3. 046, *P*=.045, reflecting an expected smaller center of mass displacement along the vertical axis for the highest impact height (IH1 2.5 cm ± 0.4 cm) versus the lowest (IH3 2.0 cm ± 0.4 cm, *P*=.024) impact height (see Figure 7).

### Success

Overall, participants had higher success rates when dodgeball was played in a first-person perspective (95.5%) versus a third-person perspective (91.5%, *F*_1,27_=5.451, *P*=.007). The success rate was also influenced by impact height (*F*_2,26_=6.018, p=.027), with participants being less successful intercepting balls at the low (IH3, 88.4% ± 2.5%) versus middle (IH2, 96.8% ± 1.0%, *P*=.005) and high (IH1, 95.3% ± 1.1%, *P*=.010) impact heights.

### NASA TLX

Avatar perspective did not affect NASA TLX score or any of its subscales (ie, mental demand, temporal demand, performance, effort, and frustration).

## Discussion

### Principal Results

The primary goal of this study was to determine the influence of avatar perspective on movement behavior. We achieved this goal by examining changes in joint excursions and center of mass displacement during virtual dodgeball gameplay in a first- and third-person perspective. Consistent with our previous study [8], a first-person avatar perspective resulted in greater joint excursions, center of mass displacement, and hand displacement at ball interception. This finding supports the notion that avatar perspective, rather than simply the mode of displaying the virtual environment and avatar (ie, HMD vs 3DTV), has the potential to influence motor behavior.

The study findings regarding the effect of avatar perspective on motor behavior are consistent with our prior work [8,9] as well an earlier study by Ustinova and colleagues [11], which found participants reached further and less accurately (by measuring the index of curvature of the finger endpoint trajectory throughout the movement) with increasing viewing angles (ie, mid-range) across third-person perspectives. Interestingly, despite these potential decrements in movement efficiency, participants reported a preference for these mid-range viewing angles compared to those with 0° curvature. This view from right behind the avatar may have obscured visual information of the avatar itself, affecting how the body is perceived in space [19] and perception of distance to reach [20]. In the current study, while we did not examine participant preference towards an avatar perspective, we did not find any significant changes in the task loads between first- or third-person perspectives (based on NASA TLX scores); however, participants were more successful and reached further in a first-person compared to third-person perspective.

The differences in joint excursions between first- and third-person perspectives could be explained by changes in how one’s avatar and environment are perceived, consistent with theories surrounding movement behavior and embodiment in first- and third-person avatar perspective during observations of avatars in virtual environments [21]. Pavone et al [21] noted that participants report a greater sense of embodiment when an avatar is observed in first- versus third-person perspective, and that embodiment declines when an observed avatar makes grasping errors while the participant is static. Further, errors in the third-person avatar perspective had lower fronto-cortical event-related potentials (triggered by performing an error) as well as medial-frontal theta power (related to action monitoring). Hence, the third-person perspective evoked less of a change in performance-related brain activity as compared to a first-person perspective, indicating errors made in the third-person avatar perspective are less perceived as being one’s own errors. However, the difference in success rates between first- and third-person avatar perspectives was significant, suggesting that in both perspectives, success rates were high (95.5% and 91.5%).Thus, avatar errors perceived from a first-person perspective are experienced with a higher sense of embodiment than those in third-person perspective [21]. Therefore, it is likely that third-person avatar perspectives negatively affect the sense of embodiment without many errors, which could be due to the less action monitoring. In the present study, although there was a significant difference in success rates between first- and third-person avatar perspectives (ie, 95.5% and 91.5%, respectively), success rates were nonetheless high in both perspectives. This suggests that a third-person avatar perspective may negatively affect embodiment without producing many errors, which could be due to the less action monitoring (medial-frontal theta power).

Additionally, avatars are perceived more like themselves in the first- than in the third-person perspective [22,23], which could also affect how reaching movements are planned. Altogether, the higher embodiment in the first-person perspective leads to higher success rates but also results in a greater center of mass displacement, and hand and joint excursions. Thus, it could be argued that the movements were more efficient when one’s avatar is presented in the third-person perspective. While addressing movement efficiency is beyond the scope of these data, the findings indicated that motor behavior could be altered by manipulating the avatar perspective, which aligns well with our previous work.

The intended impact heights of the launched virtual dodgeballs were calculated based on the participant’s trunk length, arm length, and hip height to mimic our standardized full-body reaching paradigm [13,15,16]. While lumbar flexion angles increased significantly from the highest to the lowest targets, the magnitude of lumbar flexion did not match our prior expectations based on the algorithm used to calculate individualized impact heights. Specifically, participants increased joint excursions in a compensatory fashion across several joints, particularly within the lower limb (ie, ankle, knee, hip), leveraging the kinematic redundancy inherent in these whole-body movement tasks. Similar observations have been reported previously using a standardized reaching task as a baseline for target locations [8,14,15]. Only the shoulder joint excursions did not increase across the impact heights; however, a first-person perspective did increase shoulder joint excursion as compared to a third-person perspective. Joint excursions for target reaching tasks have also been shown to be influenced by gender [14], pain-related fear [24], virtual display type [8], and comparison between VR and real-world movements [9]. In the present study, all joints but the elbow showed increased excursions in the first-person perspective as compared to the third-person perspective. The absence of an effect for the elbow joint may be due to the relatively small excursions of this joint required to successfully perform the reaching task.

Finally, the results of this study support virtual dodgeball as an effective strategy to promote lumbar flexion, thereby adding to the clinical utility of virtual dodgeball in promoting joint excursions when the avatar is presented in the first-person perspective. Additionally, similar games can be manipulated for different motions and therefore training different movement behaviors, such as targeting the center of mass motions in the antero-posterior and mediolateral planes to do balance training and kicking motions for control over leg muscle. This approach could provide benefits to populations that require increased motion to alleviate pain (eg, improving movement outcomes for those with chronic low back pain and kinesiophobia). Avatar perspective can thus drive motor behavior in VR gameplay.

### Limitations

A limitation of the present study is the absence of a direct comparison with real-world dodgeball. Of course, such a comparison would be a major challenge as real-world dodgeball would be very hard to standardize relative to VR, making it difficult to compare joint, center of mass, and limb excursions. However, simple reaching movements show differences in VR versus real-world whole body movements to the same target height [9]. Therefore it would be expected that if interception positions could be standardized, a similar increase in joint excursions would be elicited in VR compared to real-world full body in virtual dodgeball.

### Conclusions

The results of this study demonstrate that the avatar perspective can influence motor behavior. Considering that a primary goal of VR-based rehabilitation is often to restore movement following orthopedic or neurologic injury, understanding how the presentation of an avatar or, by extension, camera position will affect motor behavior is crucial in the development of VR assessment and treatment tools.

## Abbreviations

HMD: head-mounted display
TLX: Task load index
TV: television
VR: Virtual reality
3D: Three-dimensional
